# Estimation of the mortality rate of workers in Japan

**DOI:** 10.1186/s12995-022-00365-z

**Published:** 2022-12-15

**Authors:** Tatsuki Kimura, Michiya Sasaki, Takatoshi Hattori

**Affiliations:** 1 Sustainable System Research Laboratory, Central Research Institute of Electric Power Industry, 2-11-1 Iwadokita, Komae-Shi, Tokyo, 201-8511 Japan; 2 Nuclear Damage Compensation and Decommissioning Facilitation Corporation, Tokyo, Japan

**Keywords:** Mortality, Occupational risk, Regulatory standards, Workers

## Abstract

**Background:**

Risk-based decision-making is used to identify risk factors for which threshold points have not been identified. The occupational mortality rate was referred to as a reference risk. This study aimed to analyze recent trends in worker mortality using three data sources.

**Methods:**

The Reports of Worker Casualties (RWC), the Annual Statistics Report of the Industrial Accident Compensation Insurance Council (ASR), and the Annual Business Report of the Industrial Accident Compensation Insurance Council (ABR) were used. Mortality rates were estimated by age group and industry category (overall, and manufacturing and construction industries) from 1991 to 2019. The mortality rates were compared with those estimated in Western countries.

**Results:**

The mortality rates for the three industry categories in the RWC and ASR decreased, whereas those for the manufacturing and construction industries in the ABR did not. In 2019, the mortality rates in the ABR were 3.1, 4.8, and 3.1 times higher than those in the RWC overall and in the manufacturing and construction industries, respectively. The differences decreased when deaths after long-term medical treatment were considered. The mortality rate trends in Japan were similar to those in Western countries. The upper mortality rate among Japan and Western countries was at least five to 15 times higher than the lowest.

**Conclusion:**

When occupational mortality rates are used as a reference risk, it is important to consider any changes with time, the data sources used, and the differences between countries.

**Supplementary Information:**

The online version contains supplementary material available at 10.1186/s12995-022-00365-z.

## Background

Substances that have negative impacts on human are usually regulated by regulatory standards; for example, some standards refer to tolerable concentrations. These values are usually either based on scientific data or uncertainty factors if scientific data are insufficient to estimate the threshold for humans. On the other hand, for some risk factors for which threshold points have not been identified, such as carcinogens and ionizing radiation, risk-based decision-making is used. A dose–response curve and a linear no-threshold model can be used to estimate the risk. The magnitude of the estimated risk can then be used to determine the regulatory standard.

Other factors are often referred to when determining the magnitude of the risk. Deaths are often used as endpoint risk indices. The use of occupational deaths is advantageous because data on occupational deaths have been collected and recorded for a long time in many countries. From the viewpoint of risk-based decision-making, mortality rates are better than the number of deaths because the magnitude of a risk is usually described as a probability. For example, the International Commission on Radiological Protection (ICRP) estimated the annual occupational mortality rate using number of occupational accidents. The estimated result was referred to as a reference risk to determine the magnitude of risk due to ionizing radiation [3, 4].

It is beneficial to understand the trend of occupational mortality rates when they are used as a reference because mortality rates can be affected by massive disasters and accidents. In this case, it is important to consider the differences in mortality rates between industries because activities vary among them.

Because the occupational mortality rate as a reference risk used by ICRP was estimated by comparing the occupational mortality rate by category of industry in different industrialized countries, Iwasaki and co-workers [5, 6] investigated the occupational mortality rate trend by industry category in Japan from 1973 to 1990 using occupational accident data. They found a decreasing trend and inequality of occupational mortality rates by industry category. The trend of occupational accidents and diseases in Japan has also been studied [9, 10]; however, since 1991, there have been few studies about the trend of occupational mortality rates estimated by age group and industry category. Thus, it is beneficial to analyze occupational mortality rates in detail.

In this study, the trends of occupational mortality rates were estimated using three data sources obtained from 1991 to 2019 in Japan, taking into consideration that occupational mortality rates after 1991 have not been previously studied [5, 6, 9, 10]. After 2016, data were only available in ILOSTAT. Thus, mortality rates in Japan were estimated from open data related to occupational accident reports, occupational insurance claims, and the national census. In addition, the trends in Japan were compared with those in Western countries using data from ILOSTAT to better understand the trends of mortality rates in industrialized countries.

## Materials and methods

### Data

The data from three reports on occupational accidents in Japan, the Reports of Worker Casualties (RWC), the Annual Statistics Report of the Industrial Accident Compensation Insurance Council (ASR), and the Annual Business Report of the Industrial Accident Compensation Insurance Council (ABR), collected by the Japan Ministry of Health, Labour and Welfare, were used in this study (Table 1). More information on the method of collection used by these three Japanese studies is described in the appendix. The RWC is based on reports submitted to the Chief of the Competent Labour Standards Inspection Office immediately after the occurrence. These data are available in an online database (https://anzeninfo.mhlw.go.jp/, accessed February 22, 2022). There are two datasets in the RWC in 2011; data relating to the Great East Japan Earthquake of March 11 were excluded. The ASR [8] and ABR [7] are based on Industrial Accident Compensation Insurance claims. Although ASR data were used in previous studies [5, 6], the data were unavailable because the ASR was discontinued in 2000. The number of deaths is given as the number of funeral expense claims paid to the surviving family or company that holds the funeral. The number of funeral expense claims in the ABR is the total number. The number in the ASR is the number of funeral expense claims, except for cases in which medical treatment benefits were provided.

**Table 1:** Information on sources of three statistical data sources

Name	Source	Data for reference	Information
Reports of Worker Casualties (RWC)	Reports for Labour Standards Inspection Office	Number of deaths	This report must be submitted to the Chief of the Competent Labour Standards Inspection Office without delay. This value is registered in ILOSTAT
Annual Statistics Report of Industrial Accident Compensation Insurance Council (ASR)	Claims of Industrial Accident Compensation Insurance	Number of deaths	This is the number of funeral expense claims excepting claims for those who were received the medical treatment benefits
Annual Business Report of Industrial Accident Compensation Insurance Council (ABR)		Number of funeral expense claims	Funeral expense is paid to the surviving family or the company who holds the funeral

The occupational mortality rates of Western countries (United States, Germany, Netherlands, France, and the United Kingdom) were obtained from ILOSTAT (available at https://ilostat.ilo.org/, accessed February 22, 2022). The ILOSTAT data were recorded in accordance with each country’s system. EUROSTAT (available at https://ec.europa.eu/Eurostat, accessed February 22, 2022) was also used to fill in the missing data in ILOSTAT.

### Estimation of the mortality rates of workers

#### Estimation of the mortality rates of workers in Japan

Mortality rates were used as an expression of the risk of workers, in accordance with previous studies [5, 6]. The average ages at death and of the workers were estimated to account for the effect of population aging and changes in industry structure. These estimations were carried out using data obtained from 1991 to 2019, as the estimation using data from 1973 to 1990 has already been reported previously [5, 6].

To estimate the mortality rates, the national census data in Japan (available at https://www.e-stat.go.jp/en, accessed February 22, 2022) were used because no data on the number of workers by age group and category of industry were included in the RWC, ASR, and ABR. The ASR and ABR only included the total number of workers in each industry. Thus, the number of workers by age group and industry category (overall, and manufacturing and construction industries) were estimated using the national census data. The mortality rates in the RWC, ASR, and ABR were calculated using the following equations:

1 $${M}_{RWC, y, i, a}=\frac{{N}_{RWC, y, i, a}}{{W}_{C, y, i, a}}\times \mathrm{100,000}$$

2 $${M}_{ASR, y, i, a}=\frac{{N}_{ASR, y, i, a}}{{W}_{ASR, y, i}\times \left({W}_{C, y, i, a}/{W}_{C, y, i}\right)}\times \mathrm{100,000}$$

3 $${M}_{ABR, y, i, a}=\frac{{N}_{ABR, y, i}\times \left({N}_{RWC, y, i, a}/{N}_{RWC, y, i}\right)}{{W}_{ABR, y, i}\times \left({W}_{C, y, i, a}/{W}_{C, y, i}\right)}\times \mathrm{100,000}$$

where ${M}_{RWC, y, i, a}$, ${M}_{ASR, y, i, a}$ and ${M}_{ABR, y, i, a}$ are the number of deaths per 100,000 workers based on the RWC, ASR, and ABR data by age group *a* in each industry *i* in year *y*. ${N}_{RWC, y, i, a}$ and ${N}_{ASR, y, i, a}$ are the numbers of deaths based on the RWC and ASR data by age group *a* in each industry *i* in year *y*, ${N}_{ABR, y, i}$ is the number of deaths based on the ABR data in each industry *i* in year *y*, and ${N}_{RWC, y, i}$ is the number of deaths based on the RWC data by all age groups in each industry *i* in year *y*. ${W}_{C, y, i, a}$ is the number of workers based on the national census data by age group *a* in each industry *i* in year *y*, ${W}_{ASR, y, i}$ and ${W}_{ABR, y, i}$ are the numbers of workers based on the ASR and ABR data in each industry *i* in year *y*, and ${W}_{C, y, i}$ is the number of workers based on the national census data by all age groups in each industry *i* in year *y*. Furthermore, the average ages at death and of the workers were also estimated using the data from the RWC or ASR. There are six age groups in the RWC (18–19, 20–29, 30–39, 40–49, 50–59 and 60 +) and eight age groups in the ASR (–17, 18–19, 20–29, 30–39, 40–49, 50–59, 60–69 and 70 +). For the calculation of average age at deaths, the average age for each age group was considered representative, except for the highest age-group in the RWC and ASR, and for the lowest in the ASR. The average age for calculation was represented by 65 years in the RWC and 70 years in ASR for the highest age group, and was represented by 17 years in the ASR for the lowest age group. The average age in the ABR could not be estimated because no age-related data were provided in the report.

### Trend of occupational mortality rates in industrialized countries

To compare the occupational mortality rate trends of each industry in Japan with those in Western countries (United States, Germany, Netherlands, France, and the United Kingdom), the mortality rates from the Western countries were directly collected from ILOSTAT. EUROSTAT was also referred to so as to fill in any missing data in ILOSTAT.

## Results and discussion

### Estimation of occupational mortality rates of workers in Japan

**Fig. 1:**
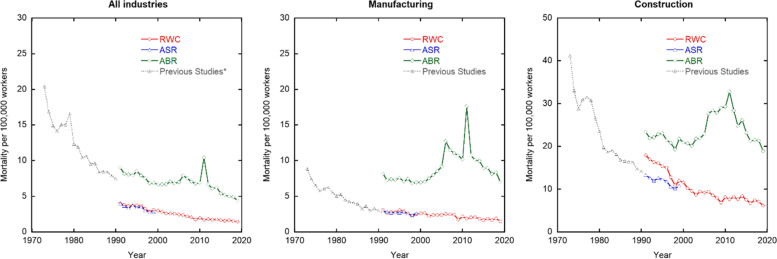
Trends of mortality rates in Japan for overall (left), and for the manufacturing (center) and construction (right) industries in the RWC, ABR, ASR and previous studies Iwasaki et al. [5] and Iwasaki and Nishizawa [6]. The symbol (*) means the values calculated using some industry’ data

The occupational mortality rates of the workers in each data sources are shown in Fig. 1. For the RWC, the mortalities per 100,000 workers were 4.0, 3.1, and 18.0 in 1991 and 1.4, 1.5, and 6.2 in 2019 overall and for the manufacturing and construction industries, respectively. The mortality rates for the three industry categories in 2019 were two to three times lower than those in 1991. The number of deaths increased in 2011 due to the Great East Japan Earthquake (March 11, 2011). These data were excluded in the RWC but not in the ABR. The ASR data trends were the same as those of the RWC data. The mortality rates overall in the ABR showed a slightly declining trend. The Ministry of Health and Labour Standards launched an occupational safety and health program in 1958 that aimed to improve the occupational environment using RWC data as the number of deaths. Thus, we would expect that the mortality rates reported in the RWC would show a slightly declining trend. In contrast, the mortality rates in the manufacturing and construction industries increased after 2000. This increasing trend may have been caused by deaths due to asbestos since the Act on Asbestos Health Damage Relief was enforced in 2006 in Japan. Furthermore, there has been a large increase in mesothelioma cases due to occupational asbestos exposure compensated after 2004 [10]. It should also be considered that the latency period of mesothelioma is about 30–40 years. The effect of the latency period on the mortality rates is discussed in another subsection. The mortality rates in 2019 in the ABR were 3.1, 4.8, and 3.1 times higher overall and for the manufacturing and construction industries, respectively. The main reason for this could be related to the rules for recording deaths. Deaths in the RWC were based on reports submitted immediately after the occurrence. In other words, deaths after long-term medical treatment for occupational diseases were not included in the number of deaths in the RWC. The number of deaths in the ASR is calculated by subtracting the number of cases in which medical treatment benefits were received for more than 18 months from the total number of funeral expense claims. The reason why the trends of mortality rates are also the same may be that the data collection rule in the RWC is similar to that in the ABR and that in most cases of death after medical treatment, medical treatment benefits have been received. In contrast, the number of deaths in the ABR is taken from the total number of funeral expense claims. Thus, the difference in mortality rates seems to be due to the number of cases in which medical treatment benefits were received, namely, the number of patient receiving long-term medical treatment. To the best of our knowledge, this is the first time that the difference between these three Japanese statistical data sources has been estimated from the point of view of mortality rates.

**Fig. 2:**
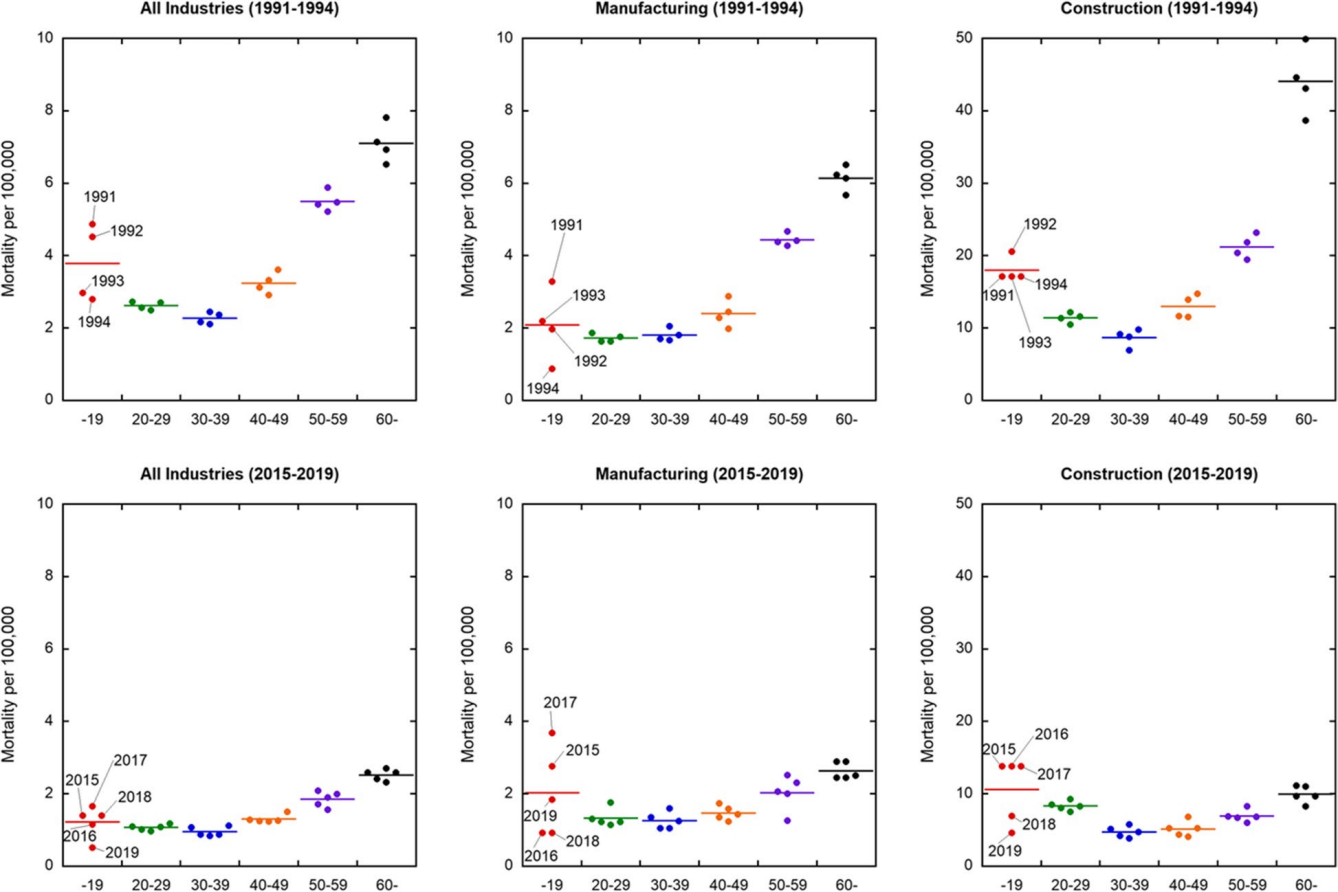
Trends of mortality rates in Japan in the RWC by age groups overall (left), and the manufacturing (center) and construction (right) industries. Upper figures show the mortality rates from 1991 to 1994 and lower figures show those from 2015 to 2019. Plots and bars mean the values of mortality rate in each single year and the average for each age group, respectively

The number of deaths per 100,000 workers in the RWC by age group and industry category is shown in Fig. 2. The mortality rates of those aged 60 + and 50–59 years were higher than those aged 30–39 and 40–49 years, and the mortality rate of those under 19 years was highly variable. This is because the number of workers under 19 years is relatively small in Japan.

**Fig. 3:**
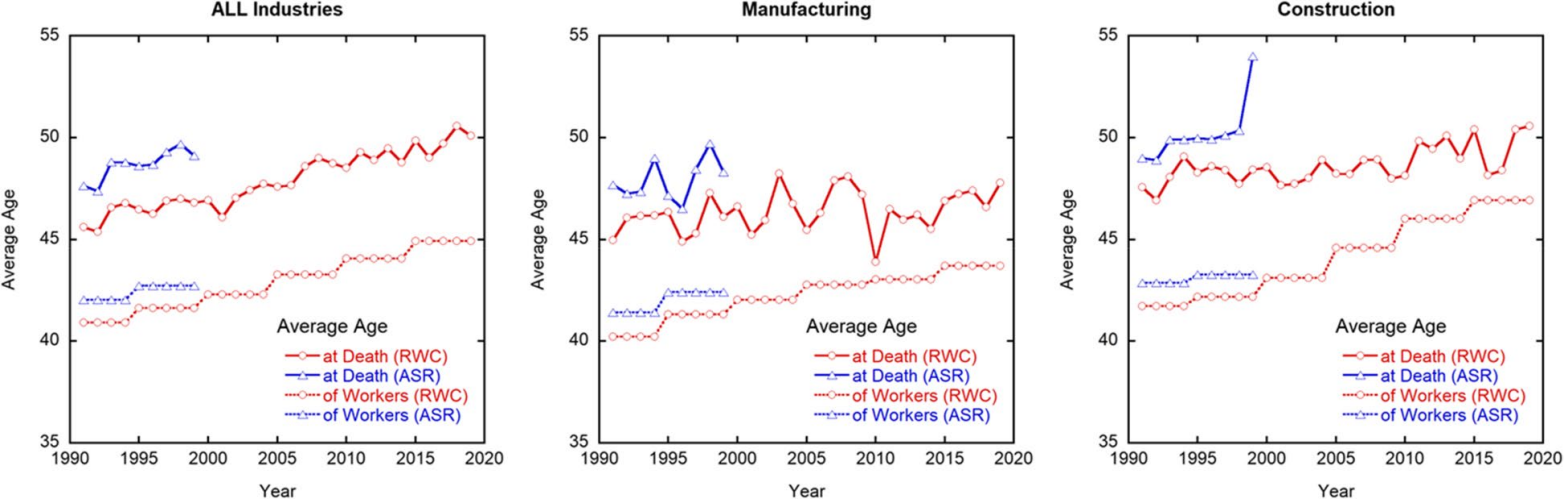
Trends of average ages at death and of workers in Japan overall (left), and the manufacturing (center) and construction (right) industries

The average ages at death and of the workers in the RWC and ASR are shown in Fig. 3. The five-year averages for the average ages at death and of the workers in Fig. 3 are shown in the Supplement (Table 2). The dashed lines for the estimated average age of the workers appear stepped because these values were estimated using data from the national census, which is conducted every five years. Both average ages overall are increasing, and the differences are almost constant from 1991 to 2019 (approximately five to six years). The average age in the manufacturing and construction industries is also increasing; however, the differences between the average ages at death and of the workers decreased to 3.5 and 2.7 years in 2019, respectively. Furthermore, the average age of the workers in the ASR is one year and two years higher than in the RWC, respectively. These differences could be due to the differences in the age group categories. In the ASR, there were two age groups of elderly workers (60–69 and 70 years and over) and younger workers (less than 17 and 18–19). However, the age group of elderly workers in the RWC is only 60 years and over, and that of younger workers is only 18–19. This explanation is consistent with our findings that when the average age at death in the ASR was recalculated using the age group categories in the RWC, the results approximately agreed with that in the RWC (Figure S1).

**Table 2:** List of five-year averages for average ages at death and of the workers

		1991–1994	1995–1999	2000–2004	2005–2009	2010–2014	2015–2019
Average age at death	All industries	46.1^a^	46.7	47.0	48.3	49.0	49.8
	Manufacturing	45.8^a^	46.0	46.6	47.0	45.6	47.2
	Construction	47.9^a^	48.3	48.2	48.4	49.3	49.6
Average age of workers	All industries	40.2^a^	41.6	42.3	43.3	44.1	44.9
	Manufacturing	40.2^a^	41.3	42.0	42.8	43.0	43.7
	Construction	41.7^a^	42.2	43.1	44.6	46.0	46.9

^a^The data are the averages from 1991 to 1994 because of the lack of data for 1990

### Occupational mortality rate trends in industrialized countries

**Fig. 4:**
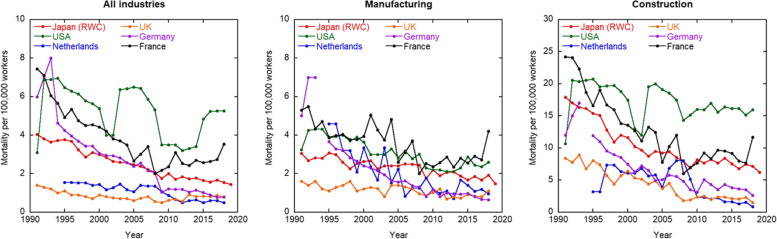
Trends of mortality rates in the United States, Germany, the Netherlands, France, the United Kingdom and Japan overall (left), and the manufacturing (center) and construction (right) industries

This study makes it possible to compare recent occupational mortality rate trends in Japan with those in Western countries. The number of deaths per 100,000 workers in Western countries (United States, Germany, Netherlands, France, and the United Kingdom) and Japan (RWC) is shown in Fig. 4 and Table S1. The mortality rates have declined over time, except for overall and those in the construction industry in the United States. The current mortality rates in Germany, the Netherlands, and the United Kingdom are lower than in other countries. The ranges of mortality rates in the last ten years (2009–2018) are roughly estimated as 0.5–5, 0.5–2.5 and 1–15 per 100,000 workers overall and for the manufacturing and construction industries, respectively. This indicates that the upper mortality rates are at least five to 15 times higher among industrialized countries than the lowest mortality rates. In 2018, the highest mortality rates overall and for the manufacturing and construction industries were 10.8, 6.5, and 18.1 times higher than the lowest rates, respectively. Further analysis of the data sources is needed to compare these countries’ mortality rates. As described above, the mortality rates in Japan differ according to the data sources. The data sources recorded in ILOSTAT also differ between countries. The records of ILOSTAT in Japan and the United Kingdom are based on labour inspection reports; in Germany, the Netherlands and France, they are based on insurance records; and in the United States, they are based on an established survey. Additionally, the data source in the United States for ILOSTAT was the Current Employment Statistics Survey until 1991 and the Census of Fatal Occupational Injuries since 1992. Interestingly, the mortality rate overall in the United States increased two-fold in 1992 (Fig. 4) due to data source change. This indicates that the differences among the reference data sources may not be unique to Japan but a problem common to other countries.

### Considerations for the possible underestimation and time lag between occurrence and certification

This study aimed to estimate the occupational mortality rates in Japan using open-source data. However, this study showed what likely affects mortality rates; underestimation and time lag.

The occupational mortality rates were calculated using statistical data related to occupational accidents. The number of reported cases tends to be underestimated because of the nature of the occupational accidents. Hämäläinen [1, 2] used a factor of 1.126 to compensate for the underestimation of fatal occupational accidents. When including deaths due to occupational diseases, Hämäläinen [1] estimated that the number of deaths due to occupational diseases in Japan was more than ten times larger than that due to occupational accidents. In this study, the mortality rates, except for those in 2011, calculated using data from the ABR were roughly 1.2 to 6.1 times higher than those calculated using data from the RWC. However, no calculation to compensate for the underestimation was performed in this study because no open data sources were identified that analyzed the number of unreported cases in Japan.

The mortality rates were defined as the ratio of deaths to the number of workers. When the number of deaths is estimated from insurance claims, a time lag between the occurrence of occupational accidents and the certification of claims is inevitable. In this study, the mortality rates from the ABR were particularly affected because the mortality data from the ABR included deaths due to occupational asbestos exposure, which has a long latency.

The results of this study should be interpreted in consideration of these factors. Occupational mortality rates can be used as a reference to determine the magnitude of risk. However, the estimation results of occupational mortality rates differ according to the data source, whether labor inspection reports or insurance records are used, and the method of collection.

## Conclusions

This study analyzed the mortality rates of workers in Japan from 1991 to 2019 using three data sources (RWC, ASR, and ABR). The mortality rate trends by age group and three industry categories (overall, and manufacturing, and construction industries) and those of the average ages at death and of the workers were clarified. Furthermore, the differences between the data sources were revealed. The trends of the mortality rates in the RWC and ASR declined, but those in the ABR for the manufacturing and construction industries did not. The mortality rate in the ABR tended to be higher than that in the RWC. In 2019, the mortality rates in the ABR were 3.1, 4.8, and 3.1 times higher than those in the RWC overall and for the manufacturing and construction industries, respectively. These differences are related to the number of deaths among workers who had undergone long-term treatment for occupational diseases. In addition, it was revealed that the average age at death and of the workers were increasing overall and for the manufacturing and construction industries. The differences between both average ages were almost constant for all industries and became smaller for the manufacturing and construction industries.

The mortality rate of the RWC in Japan was compared to that in Western countries. The trend of the RWC in Japan was similar to that in other countries. The upper mortality rates were found to be at least five to 15 times higher than the lower mortality rates among industrialized countries. It should be noted that mortality data cannot be compared precisely because it is collected inconsistently.

Regulatory standards are implemented based on various societal and practical factors. It should be noted that the mortality rate of workers is just one aspect that is considered when deciding on regulatory standards. When the occupational mortality rate is used as a reference, it is important to consider the changes over time, the data sources used, and the differences between countries.

## Supplementary Information

**Additional file 1.** Recalculation of average age at death for the ASR using the age group categories for the RWC

**Additional file 2.** List of mortalities per 100,000 workers in each country in 1991 and 2018

**Additional file 3.** Information on the three statistical data sources

## Data Availability

The datasets used and analysed during the current study are available from the corresponding author on reasonable request.

## Abbreviations

RWC: Reports of Worker Casualties
ASR: Annual Statistics Report of Industrial Accident Compensation Insurance Council
ABR: Annual Business Report of Industrial Accident Compensation Insurance Council
