# Supplementary material for: Estimation of the mortality rate of workers in Japan
Source: J Occup Med Toxicol. 2022 Dec 15;17:24. doi: 10.1186/s12995-022-00365-z (PMC9753261; doi:10.1186/s12995-022-00365-z)
Supplement: Supplementary file 1 — Additional file 1. Recalculation of average age at death for the ASR using the age group categories for the RWC [file 12995_2022_365_MOESM1_ESM.docx]

Supplementary Material (for publication)


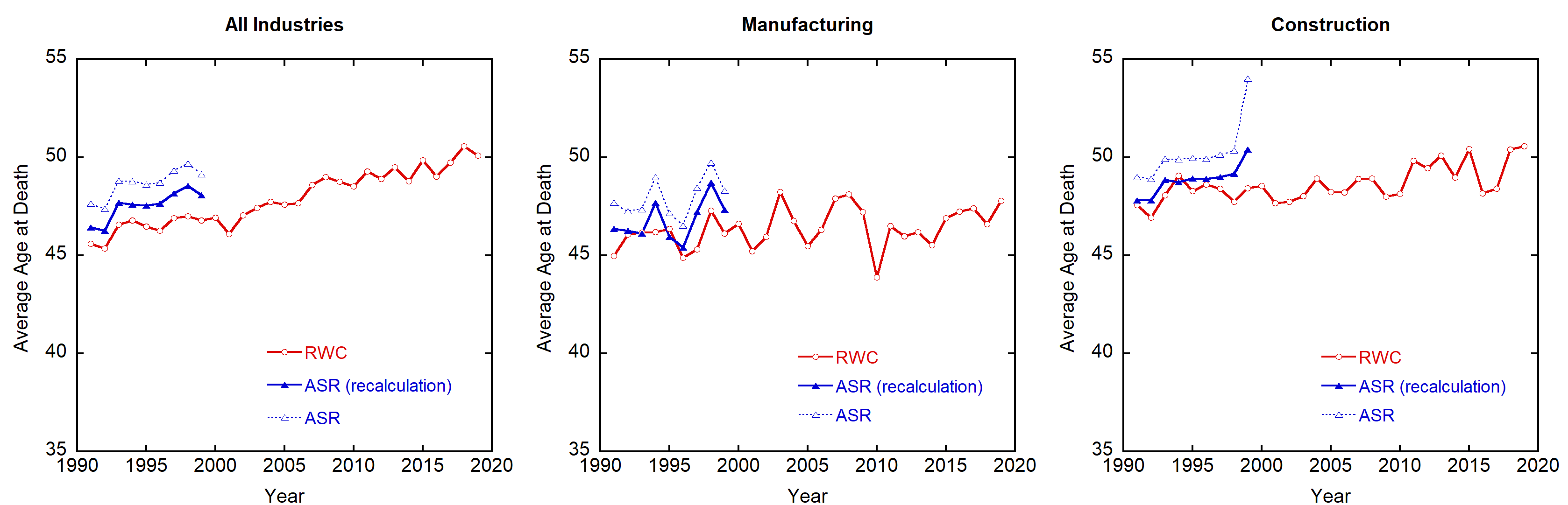
**Supplementary Fig. S1.** Recalculation of average age at death for the ASR using the age group categories for the RWC. The recalculated average age for ASR data is almost the same as that for RWC data for manufacturing and construction industries, and the difference is smaller for all industries.
