# Supplementary material for: Estimation of the mortality rate of workers in Japan
Source: J Occup Med Toxicol. 2022 Dec 15;17:24. doi: 10.1186/s12995-022-00365-z (PMC9753261; doi:10.1186/s12995-022-00365-z)
Supplement: Supplementary file 2 — Additional file 2. List of mortalities per 100,000 workers in each country in 1991 and 2018 [file 12995_2022_365_MOESM2_ESM.docx]

Supplementary Material (for publication)

**Supplementary Table S1.** List of mortalities per 100,000 workers in each country in 1991 and 2018

| Country | All industries | | Manufacturing | | Construction | |
| --- | --- | --- | --- | --- | --- | --- |
|  | 1991 | 2018 | 1991 | 2018 | 1991 | 2018 |
| U.S. | 3.1 | 5.3 | 3.3 | 2.6 | 10.7 | 16.0 |
| Netherlands | 1.7^a)^ | 0.5 | 1.5^a)^ | 1.0 | 4.1^a)^ | 0.9 |
| U.K. | 1.4 | 0.8 | 1.6 | 1.1 | 9.4 | 1.5 |
| Germany | 6.0 | 0.8 | 5.0 | 0.6 | 12.0 | 2.6 |
| France | 7.4 | 3.5 | 5.3 | 4.2 | 24.2 | 11.7 |
| Japan (RWC) | 4.0 | 2 | 3.1 | 2 | 17.9 | 9 |
| Average | 3.9 | 2.2 | 3.3 | 1.9 | 12.9 | 7.0 |

^a)^The data for Netherlands in 1990 is lacking. This value is for 1989.
