# Supplementary material for: Estimation of the mortality rate of workers in Japan
Source: J Occup Med Toxicol. 2022 Dec 15;17:24. doi: 10.1186/s12995-022-00365-z (PMC9753261; doi:10.1186/s12995-022-00365-z)
Supplement: Supplementary file 3 — Additional file 3. Information on the three statistical data sources [file 12995_2022_365_MOESM3_ESM.docx]

Information on the three statistical data sources

The Reports of Worker Casualties (RWC) is based on reports from the Labour Standards Inspection Office. According to Article 97, Ordinance on Industrial Safety and Health, “when a worker has died or taken absence from work due to industrial accident or injury, suffocation or acute poisoning during work, or within the workplace or building attached thereto, the employer must submit a report using Form No. 23 to the Chief of the Competent Labour Standards Inspection Office without delay” (https://www.japaneselawtranslation.go.jp/ja/laws/view/3878#je_pt1ch13at35). These reports are used to understand the occurrence of occupational accidents and prepare plans for their prevention. The Ministry of Health, and Labour Standards collects reports that are considered occupational accidents and publishes them in the RWC every year.

The Annual Statistics Report of the Industrial Accident Compensation Insurance Council (ASR) (*roudoushasaigai hoshohoken roudousaigai toukei nenpou*) and the Annual Business Report of the Industrial Accident Compensation Insurance Council (ABR) (*roudoushasaigai hoshohoken zigyou nenpou*) are based on Industrial Accident Compensation Insurance claims. The Labour Standards Bureau of the Ministry of Health, and Labour Standards, the prefectural Lobour Bureau and the Labour Standards Inspection Office uses data on the insurance benefits claimed. The Ministry of Health, and Labour Standards publishes the ASR or ABR annually using this data.


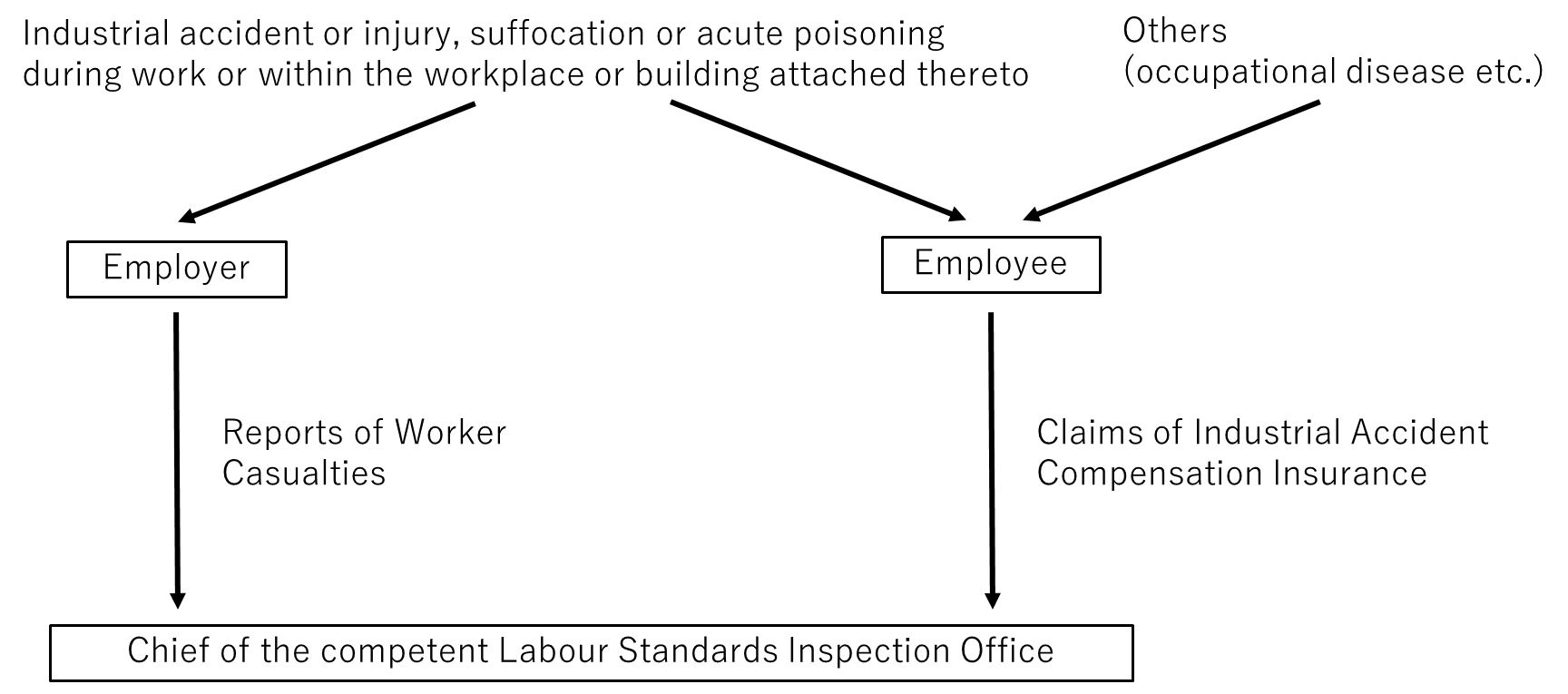


Fig. The way of data collection
